# Rehabilitation Management of Transcervical Neck Femur Fracture and Segmental Tibia Fracture: A Case Report

**DOI:** 10.7759/cureus.25902

**Published:** 2022-06-13

**Authors:** Vishnu R Bhure, Shivani R Uttamchandani, Pratik Phansopkar

**Affiliations:** 1 Physical Therapy, Datta Meghe Institute of Medical Sciences, Wardha, IND; 2 Musculoskeletal Physiotherapy, Datta Meghe Institute of Medical Sciences, Wardha, IND; 3 Musculoskeletal Physiotherapy, Ravi Nair Physiotherapy College, Datta Meghe Institute of Medical Sciences, Wardha, IND

**Keywords:** trauma and orthopedics, postoperative physiotherapy intervention, physiotherapy rehabilitation, physiotherapy interventions, physiotherapy

## Abstract

In the elderly population, the proximal femoral fracture is a major health concern. Surgical treatment of this fracture, combined with postoperative physical therapy, is used to reduce morbidity. The primary goal of this study was to investigate tibial and femoral neck fractures. It was managed by physiotherapy post-operatively and had the patient perform activities of daily living with no resistance. In this case, a 45-year-old male patient was traveling when he was involved in a traffic accident, causing injury to his left lower limb. He was operated on with open reduction and internal fixation with a tibia interlocked nail for a segmental tibia fracture on the left side, as well as cannulated screw fixation for a femoral neck fracture. Physiotherapy management was done, focusing on his occupational needs and rehabilitation for the betterment of activities of daily living.

## Introduction

Hip fractures are a common injury in the emergency room, especially in the elderly and also seen in young patients who participate in sports or suffer from high-energy trauma [1]. According to the literature, femoral neck fractures in young adults are most commonly caused by high-energy trauma such as car accidents [2]. To avoid life-threatening joint complications, prompt diagnosis and treatment are required [3].

Intracapsular femoral neck fractures are common in the elderly after a minor fall [4]. Femoral neck fractures in adults under the age of 50, on the other hand, are uncommon and frequently the result of high-energy trauma [5]. The biomechanical difficulties of femoral neck fixation, combined with the vulnerability of the femoral head blood supply, resulted in a high incidence of nonunion and osteonecrosis of the femoral head (ONFH) following internal fixation of displaced femoral neck fractures [6]. The most common pattern is a Pauwels type III fracture [7,8]. The first biomechanical classification for femoral neck fractures, the Pauwels classification, is still used today [9].

A tibial segmental fracture is a relatively rare injury. This type of fracture is most commonly associated with significant soft tissue injury, long-term impairment, and physical disability following high-energy trauma [10]. Treatment for segmental tibial fractures is difficult, and the technique used for initial fracture stabilization is controversial, yielding frequently unsatisfactory results [11]. Patients with these injuries are frequently referred to outpatient physical therapy (OPT) after receiving acute injury management. Physical therapy improves clinical outcomes such as range of motion (ROM), gait speed, and function [10].

The patient's femoral neck fracture was treated with open reduction internal fixation with tibia interlock nailing and cannulated screw fixation for proximal tibia fracture in this case report. Rehabilitation protocols vary depending on the case and type of fracture, the surgeon, physical therapist, and clinical experience. 

## Case presentation

Patient description

A 45-year-old male patient, a carpenter by profession, met a road traffic accident when he was traveling on his motorcycle and a two-wheeler came from the wrong side and collided with him sustaining an injury to his head, left leg, and eye. Immediately after the fall, he experienced sudden pain in his left lower limb, which was so severe (7/10 on the numerical pain rating scale) that he was unable to stand on his feet. The patient was aware, oriented to place and time, and managed to bring to Acharya Vinoba Bhave Rural Hospital, Sawangi Meghe, Wardha, with complaints of hip and knee joint pain and difficulty performing any actions. 

MRI and USG examinations revealed a transcervical neck femur fracture and a segmental tibia fracture on the left side. He was operated on with open reduction and internal fixation (ORIF) with tibia interlocked nail for segmental tibia fracture and cannulated screw fixation of femoral neck fracture on November 20, 2021 with the above-knee slab. The patient was referred to physiotherapy with complaints of intense pain in the left lower limb and difficulty performing movements of the left lower extremity from the hip joint. The pain was sharp and shooting; the onset was sudden, aggravated by movement, and relieved by rest; and the intensity of pain on a numerical pain rating scale was 9/10. Table 1 shows the timeline of the events. 

**Table 1:** Timeline.

Occurrences	Dates
Date of injury	19/11/21
Date of surgery	20/11/21
Starting of rehab protocol	23/11/21
Date of cast removal	25/12/21

Clinical findings

After obtaining the patient's consent, he was examined for clinical and radiological outcomes as well as complications. On general examination, the patient appeared to be awake, well-oriented in terms of time, location, and person, and cooperative. The patient was hemodynamically stable and afebrile, with a blood pressure of 129/76 mm Hg, a pulse rate of 74 beats per minute, and a respiratory rate of 19 breaths per minute. There were no signs of cyanosis, icterus, clubbing, or edema in the patient. During the examination, the patient's left leg was gently raised on a pillow, and the temperature was normal when palpated. There was swelling across the knee and ankle. Tenderness of grade 2 was found on bony landmarks below the knee. Muscular strength is explained in Table 2 by manual muscle testing. 

**Table 2:** Manual muscle testing during the treatment.

Hip	Post-op. week 1		Week 2		Week 3	
	Right Left		Right Left		Right Left	
Flexor	3/5	2/5	4/5	2/5	5/5	3/5
Extensor	4/5	1/5	4/5	2/5	5/5	3/5
Adductor	5/5	2/5	5/5	2/5	5/5	3/5
Abductor	5/5	2/5	5/5	2/5	5/5	3/5
Medial rot.	5/5	2/5	5/5	2/5	5/5	3/5
Lateral rot.	5/5	1/5	5/5	2/5	5/5	3/5
Knee						
Flexor	4/5	0/5	3/5	2/5	5/5	3/5
Extensor	4/5	0/5	4/5	2/5	5/5	3/5
Ankle						
Dorsiflexor	5/5	3/5	5/5	3/5	5/5	4/5
Plantar flexor	5/5	3/5	5/5	3/5	5/5	4/5
Inversors	5/5	2/5	5/5	4/5	5/5	4/5
Eversors	5/5	2/5	5/5	4/5	5/5	4/5
Foot						
Flexors	5/5	3/5	5/5	3/5	5/5	4/5
Extensors	5/5	3/5	5/5	3/5	5/5	4/5

Pre-operative radiological Impression

On both limbs, range of motion (Table 3) and patients' activity limitations and participation restrictions (Table 4).

**Table 3:** Pretreatment range of motion of the affected limb (left). ROM: range of motion.

	Pre-treatment ROM		Treatment week 1			Treatment week 3			Treatment week 6		Treatment week 9		
	Active ROM	Passive ROM	Active ROM	Active ROM	Active ROM		Passive ROM	Passive ROM		Passive ROM		Active ROM	Passive ROM
HIP joint													
Flexion	20°	40°	25°	25°	25°		50°	70°		90°		90°	120°
Extension	20°	30°	25°	25°	25°		35°	60°		65°		70°	75°
Abduction	Painful	Painful	10°	10°	10°		15°	25°		45°		50°	60°
Internal rotation	Restricted	Restricted	5°	5°	10°		15°	15°		25°		20°	30°
External rotation	Restricted	Restricted	5°	5°	10°		15°	15°		25°		20°	30°
Knee joint													
Flexion	Restricted	Restricted	10°	10°	30°		35°	60°		70°		80°	85°
Extension	Restricted	Restricted	10°-0°	10°-0°	30°-0°		35°-0°	60°-0°		70°-0°		80°-0°	85°-0°
Ankle joint													
Plantar flexion	10°	15°	10°	10°	20°		30°	25°		35°		30°	35°
Dorsiflexion	5°	6°	8°	8°	15°		25°	20°		25°		25°	30°

**Table 4:** Showing activity limitations and participation restrictions based on the International Classification of Functioning.

Activities/participations			Independent	Assisted	Impossible	Comment
Mobility						
(1)	Walking		X	X	🗸	Patient was unable to perform the activity.
(2)	Squatting		X	X	🗸	Patient was unable to perform the activity.
(3)	Stairs		X	X	🗸	Patient was unable to perform the activity.
(4)	Running		X	X	🗸	Patient was unable to perform the activity.
Transfers						
(1)	Lie to sit (and opposite)		X	🗸	X	Patient was able to perform the activity under assistance.
(2)	Sit to stand (and opposite)		X	🗸	X	Patient was able to perform the activity under assistance.
(3)	Stand to the floor (and opposite)		X	🗸	X	Patient was able to perform the activity under assistance.
(4)	Sit to sit		X	🗸	X	Patient was able to perform the activity under assistance.
Balance						
(1)	Sitting		X	🗸	X	Patient was able to perform the activity under assistance.
(2)	Standing		X	X	🗸	Patient was unable to perform the activity.
(3)	On one leg		X	X	🗸	Patient was unable to perform the activity.
Upper limb functions						
(1)	Grasp	R	🗸	X	X	
		L	🗸	X	X	
(2)	Release	R	🗸	X	X	
		L	🗸	X	X	
(3)	Fine manipulation	R	🗸	X	X	
		L	🗸	X	X	
(4)	Holding	R	🗸	X	X	
		L	🗸	X	X	

**Figure 1:**
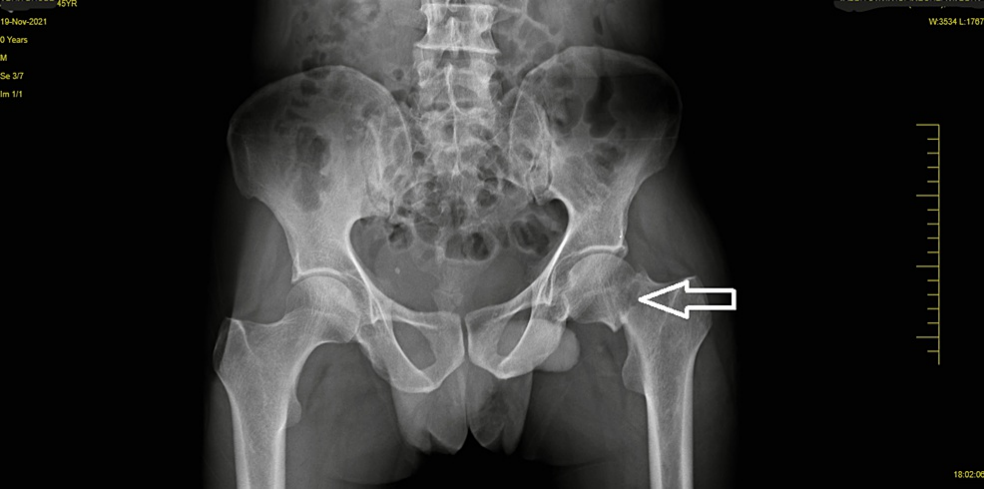
X-ray of the hip (AP view) shows a fracture of the femoral neck. AP view: anteroposterior.

**Figure 2:**
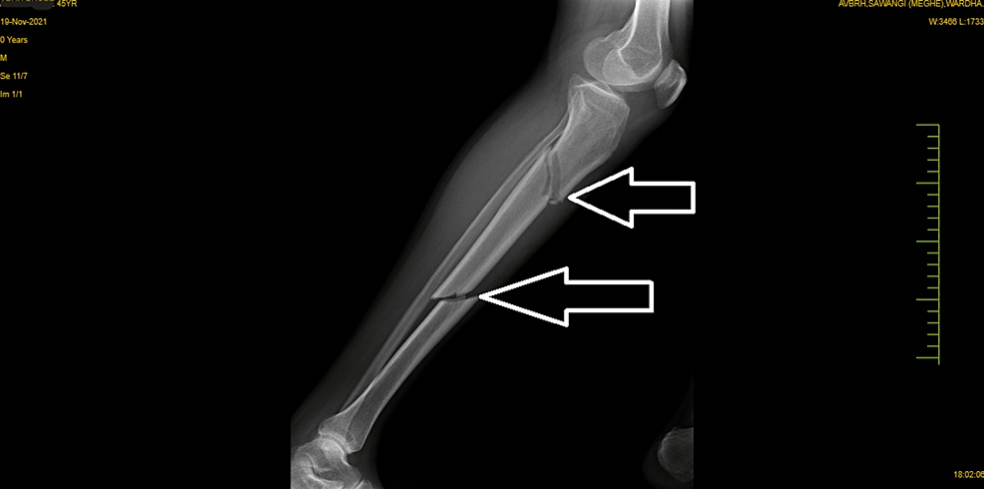
X-ray tibia (lateral view) shows a tibial shaft fracture.

**Figure 3:**
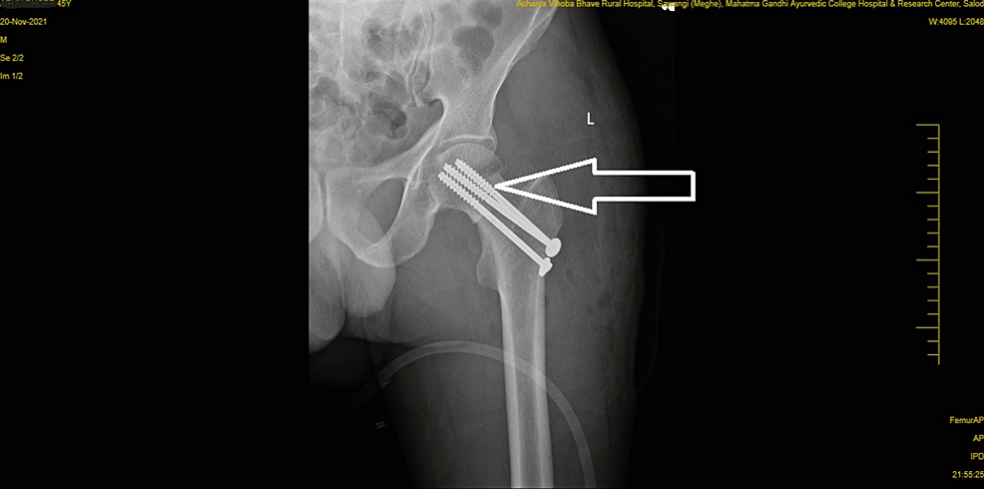
Showing cannulated screw fixation of a femoral neck fracture.

**Figure 4:**
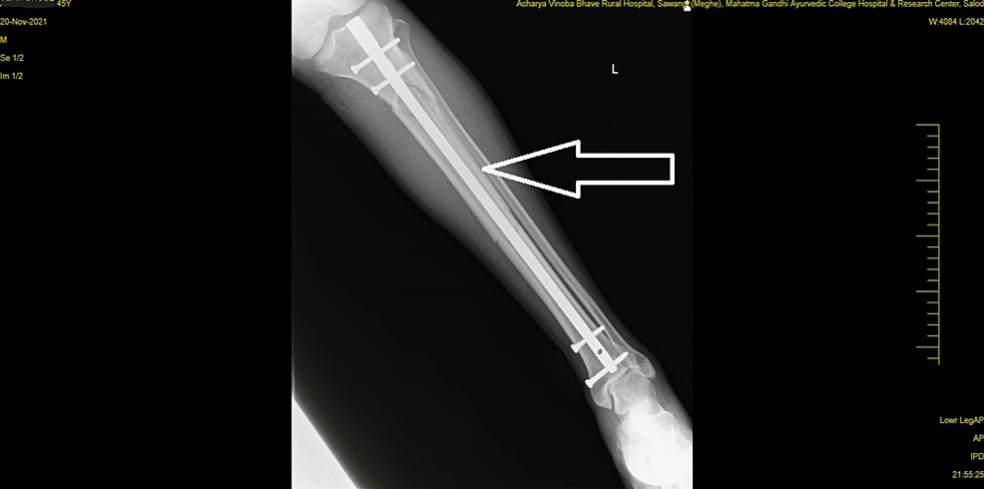
Postoperative (PA view) x-ray of the tibia showing interlocking. PA: posteroanterior.

**Figure 5:**
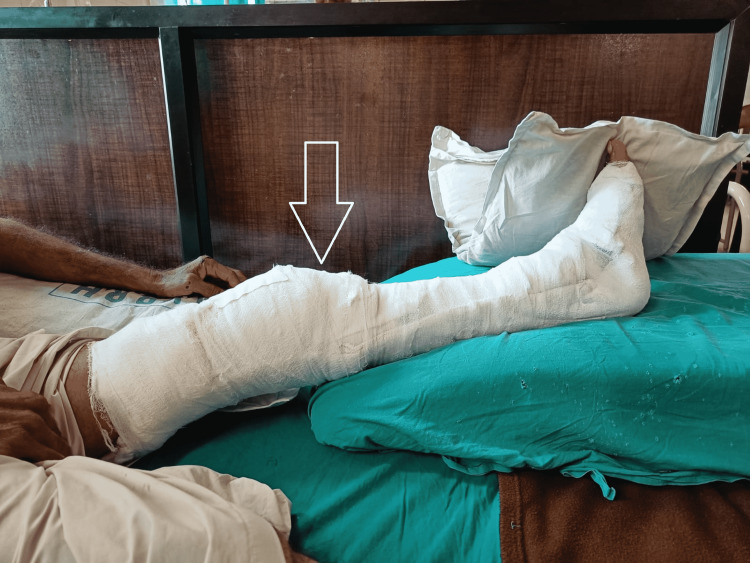
Affected lower limb in elevation.

A visual analog scale (VAS) was used to assess clinical performance; the patient scored 7 out of 10 at rest on the affected site, and a resisted isometric contraction for hip and knee flexion grading was 1 (weak and painful). Figure 1 and Figure 2 show pre-operative x-rays, and Figure 3 shows cannulated screw fixation of a femoral neck fracture. Figure 4 shows the postoperative posteroanterior (PA) view x-ray of the tibia showing interlocking. Figure 5 shows the affected lower limb in a plaster cast in elevation.

Therapeutic rehabilitation management is shown in Table 5. 

**Table 5:** Rehabilitation management.

Phase (week-wise) goal	Therapeutic exercise
Week 1	During the immobilization phase-14 days (November 23, 2021 to December 7, 2021). Chest physiotherapy was administered, including deep breathing, pursed-lip breathing, and thoracic expansion exercises. Checks were made regularly to verify that an appropriate posture was being maintained. The broken limb was subjected to 10 repetitions of resistive ankle-toe movement. Isometrics with 10 repetitions, three sets a day, holding for 10 seconds each, for quadriceps, hamstrings, hip extensors, and abductor's muscles. Isometrics to both the gluteus maximus and the medius were performed with maximum second hold and intense muscle contractions.
To reduce pain (weeks 1 and 2)	Cryotherapy, application of an ice pack on the painful area for 8-10 minutes.
During week 3	During mobilization (December 8, 2021 to December 14, 2021). In week 3, the patient sits in bed with the affected limb supported. Weight shifts were performed bilaterally with five repetitions, three sets a day. A walker was used to provide guided walking training. To avoid pain, gradual partial weight-bearing of 25% of body weight was initiated with suitable support. Stitches had been removed by this point, and soft tissue healing had progressed sufficiently. Passive relaxing motions, such as pursed-lip breathing and progressive muscle relaxation of the operated leg, were started in the initially available range. In addition to this, a controlled, pain-free continuous passive motion was started. Continuous passive motion apparatus was utilized to begin relaxed passive hip and knee flexion-extension. Self-aided dragging of the heel was used to create a progressive assisted active range of motion. Self-assisted sitting and transferring with legs dangling over the edge of the bed. The patient's normal leg supports the operated leg. Sling suspension and self-aided technology were used to start the assisted abduction, flexion, and extension. Despite some discomfort, self-assisted straight leg raises were started early.
During weeks 4-8	Hip flexion had reached 90 degrees at this point (December 15, 2021 to January 11, 2022). From week one to week three, all exercises were continued with increased repetitions. A weight cuff was used to perform dynamic quadriceps at the edge of the bed more resistant. Progressive resistance workout techniques were used to strengthen the glutei and quadriceps muscles. Passive glutei and quadriceps stretching was administered and proceeded to achieve complete ROM at the hip and knee joints.
Week 8-16	All exercises were intensified to maximal contractions (from 15 seconds hold to 30 seconds hold) in both the legs and the greatest range of motion with increased resistance.
By the week 16-18	Week 16 is the start of full weight-bearing. Spot marching and weight-bearing movements were used to kick off this program.
Correction of the limp	To maintain normal posture, a repetitive session of mirror activity in self-resistance exercise and gait training with adequate footwear is ideal. Excessive inclination to move the affected limb into adduction and internal rotation in mirror walking are necessary to avoid susceptive tendencies to fall. It's a common occurrence with open reduction internal fixation patients.

## Discussion

The most common type of tibial shaft fracture after high-energy direct trauma is a segmental fracture. In this case, the patient complains of left lower limb pain and swelling. The primary goals of rehabilitation were to reduce pain and tenderness, improve ROM and muscle strength in the patient's left lower limb, and restore the patient's functional activities at home and work.

To begin physical therapy, we started with the static quadriceps, hamstrings, and glutei. We focused on gaining functional mobility and reducing joint contracture when the patient was in bed by performing ROM exercises. The professional must be familiar with the type of fracture as well as the material used for surgical fixation. These data will interfere with the behavior, which includes walking time, weight-bearing on the limb, and, in some cases, movement restrictions.

The rehabilitation protocol was broken down into four stages. For five weeks, the patient's left lower limb was immobilized in a plaster cast to reduce tension and realignment of the femur and tibia. During the immediate postsurgical phase (one to three weeks), the patient was instructed to avoid active movement and weight-bearing on the affected limb.

During the early phase of rehabilitation, we majorly focused on unaffected limb strength and gaining functional mobility in the upper limb and unaffected lower limb. In this study, we also focused on bedside mobility during the immobilization phase.

Internal fixation with intramedullary nailing has a high rate of union for segmental tibial fractures. A significant number of these patients do not regain their pre-fracture functional level. Less than half of the survivors can walk without assistance a year after surgery, and only 40% can perform independent activities of daily living [10].

All exercises were focused on aerobic fitness and were added to the treatment plan. It showed the result of improvement in the patient's physical function because cardiorespiratory fitness increased walking capacity [10].

We should also consider an important factor that can influence our treatment, which is pain. This can cause a delay in recovery, and a high level of pain in the postoperative period has been linked to a longer hospital stay, lower adherence to physiotherapy treatment protocols, and decreased walking capacity up to three days after the procedure [11].

## Conclusions

The most common type of femoral fracture is a neck femur fracture, which can be treated surgically with the right rehabilitation protocol to avoid further malformation. In this case, during eighteen weeks of rehabilitation, there was a great improvement noticed stepwise as expected, includes of good muscle strength, reduction of pain, preventing of further complications, in gait, and allowing a return to daily activities. This case report lays out a well-organized and comprehensive rehab program for the tibial shaft and femoral neck fractures. The above case report concludes that a traditional surgical approach combined with prompt structured physiotherapy rehabilitation led to progressive improvement in functional goals, which is an important factor in achieving a successful recovery in such post-operative patients.
